# High throughput drug profiling

**DOI:** 10.1155/S1463924600000304

**Published:** 2000

**Authors:** Michael Entzeroth, Béatrice Chapelain, Jacques Guilbert, Valérie Hamon

**Affiliations:** CEREP SA 260 Boulevard St. Germain Paris 75007 France

## Abstract

High throughput screening has significantly contributed to advances in drug discovery. The great increase in the number of samples screened has been accompanied by increases in costs and in the data required for the investigated compounds. High throughput profiling addresses the issues of compound selectivity and specificity. It combines conventional screening with data mining technologies to give a full set of data, enabling development candidates to be more fully compared.


### High throughput drug profiling

**Michael Entzeroth, Béatrice Chapelain, Jacques Guilbert and Valérie Hamon**

*CEREP SA, 260 Boulevard St. Germain, 75007 Paris, France*

*High throughput screening has significantly contributed to advances in drug discovery. The great increase in the number of samples screened has been accompanied by increases in costs and in the data required for the investigated compounds. High throughput profiling addresses the issues of compound selectivity and specificity. It combines conventional screening with data mining technologies to give a full set of data, enabling development candidates to be more fully compared.*

### Introduction

With the development of high throughput screening (HTS) during the past two decades, new technologies have gained access to chemistry and biology laboratories. The use of both laboratory robotics and automated workstations has greatly increased the number of chemical entities that are synthesized for and tested against new targets. While a decade ago a daily throughput of about 1 000 compounds was considered sufficient, nowadays screening laboratories aim to achieve 100 times as many samples in the same time. Combinatorial chemistry has increased the daily output comparably and HTS has become an important success factor for early lead finding. Nearly all drug discovery research projects in the pharmaceutical industry employ HTS screening assays as initial steps to discover the chemical leads. These compounds provide the structural basis for further medicinal chemistry activities that focus on optimization of the lead compounds with respect to the activity and selectivity profile in order to identify the development candidate.

The pace of this technological development not only has created benefits such as shortening the time required for lead identification but also has generated other considerable issues. Among these are the extensive cost increases with the increased number of samples investigated. Assay miniaturization and related developments have helped to reduce the impact. The number of chemical entities that enter the primary screening and the resultant number of hits that require further evaluation have questioned the hit selection and profiling. HTS, with a hit rate of between 0.01 and 1%, depending on the target and test concentration used, produces several hundred primary hits while previously only a handful of suitable candidates were available. The decision as to which candidate to follow has a direct impact on the success rate during the further development phase. The generation of primary hits and the selection of leads based on just their structural properties does not predict the probability of converting the lead into a drug. The drop-out rate during development is still considerable and is associated with high financial losses in the pharmaceutical industry. Only one out of approximately ten potential drug candidates entering phase I of development will finally reach the market. Insufficient pharmacological and pharmaceutical properties represent the majority of reasons for failure [1]. Information on the compound properties relevant for development is often incomplete with respect to data on selectivity, solubility, pharmacokinetics and toxicology, at the time when it is needed for decision making, since such data are gathered sequentially.

### High throughput profiling

With these specific issues in mind CEREP has built recently an integrated drug discovery platform in order to serve the changing and challenging needs in lead finding. The activities are based on the company's strengths such as combinatorial chemistry, molecular modelling and compound profiling. The starting point of discovery programs is the utilization of diverse combinatorial libraries, such as Odyssey 5000, to initiate the lead finding process. This library, constructed from more than 1 500 unique monomeric building blocks, was designed to identify efficiently initial leads in a new research program. The leads identified are passed on to the key component of CEREP's platform, high throughput profiling (figure 1).

High throughout profiling consists of two major components, one of which is the pharmacological profiling that addresses the issues of compound selectivity and specificity. Selectivity is determined by testing on closely related targets such as different subtypes of the target receptor (or ion channel), different isozymes of the target enzyme, or other mechanistically related targets. In most cases it is desirable to have greater than ten-fold selectivity for the desired target.

The primary purpose of specificity testing is to identify 'promiscuous' compounds that interact with several unrelated targets. Such interactions can be indicative of *in vivo* side effects or safety problems. For these reasons the selection of assays in a specificity panel is often tailored to check for known undesirable interactions.

The current standard test panel consists of a variety of receptors, channels, transporter systems and enzymes but may be easily extended according to the scientist's needs. The activity pattern obtained allows the scientist to distinguish favourable and adverse drug properties with respect to its biological activity. Table 1 gives an overview of the different assay classes selected and the number of targets in each of them.

Traditionally the pharmacological and pharmaceutical criteria have been assessed in a sequential manner with the major focus on the compound potency and selectivity. Often there is a round of compound optimization through medicinal chemistry between each step. Each

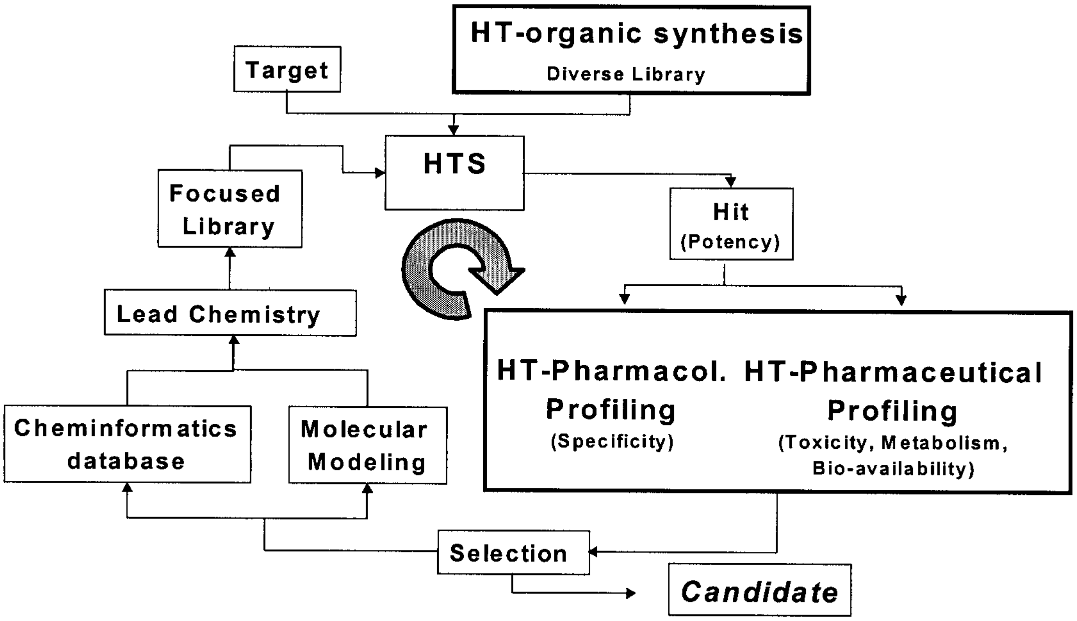

*Figure 1. Within the discovery platform at CEREP high throughput organic synthesis and high throughput profiling are main features that enable the scientist to identify the respective candidate based on a broad resource of biological results.*

*Table 1. Standard target pattern in high throughput pharmacological and pharmaceutical profiling. User-defined modification may be accommodated.*

| *Target* | *Number of assays* |
|---|---|
| Pharmacological profiling | |
| G protein coupled receptors | 29 |
| Ion channel coupled receptors | 2 |
| Transporters | 3 |
| Ion channels | 4 |
| Enzymes | 9 |
| Pharmaceutical profiling | |
| Drug Metabolism | 9 |
| Intestinal permeability | 3 |
| Drug safety | 10 |
| Solution properties | |

step has been used to rank a group of hits from primary screening. As it has not been practical to advance all compounds, the lowest ranked are generally dropped at each step. As a consequence, after significant time and expense many compounds are found to have high potency but display only poor specificity or pharmaceutical properties. The focus of the pharmaceutical profiling, as performed presently, addresses issues of solution properties, metabolism, intestinal permeability, and safety.

Now it is becoming practical through the application of HTS technologies to secondary assays to quickly and cost effectively generate a full data set encompassing the criteria above on each hit compound from primary screening. The advantage of this parallel (as opposed to sequential) approach is that key decisions are made with a full awareness of the positive and negative attributes of each compound or compound class.

An example of compound profiling is given in figure 2. The comparison of the activity pattern of the drugs investigated allows us to chose the preferred candidate out of a list of other competitors. The broad spectrum of information obtained is the basis for and represents the first step towards a knowledge-based decision in the drug discovery. The risk of taking a compound into development can be calculated and costly failures of losing a drug on its long way to an investigational new drug (IND) may be reduced or even completely avoided.

Recently we have profiled several hundred publicly available drugs and more than 100 000 data points have been collected using this approach. This has been made possible by a proprietary assay technology implemented on Zymark robot systems. In addition to the biological data sets, structural fingerprints were established using software packages that represent the spatial orientation and distance of pharmacophoric groups within the molecules. Together with the biological results they are stored in a database. The data acquired accordingly represent a unique basis for data mining with respect to both the clustering of compounds and biological tests. With the increase in the information content, it will be possible to group development candidates according to their structure with compounds of known profile in the database. With the increasing amount of information acquired, certain predictions may be possible not only with respect to the compound's activity and physicochemical profile but also with respect to its *in vivo* properties. Based on the knowledge obtained, the selection of a drug candidate with an increased probability of passing through development and reaching the IND filing may be achieved.

Another approach to using the indicative potential of the data collection is the development of focused libraries for screening against new targets. Analysis of the biological targets and the activity pattern of the drugs obtained in profiling allows the design of a test set of compounds. This may be either based on the selection of chemical

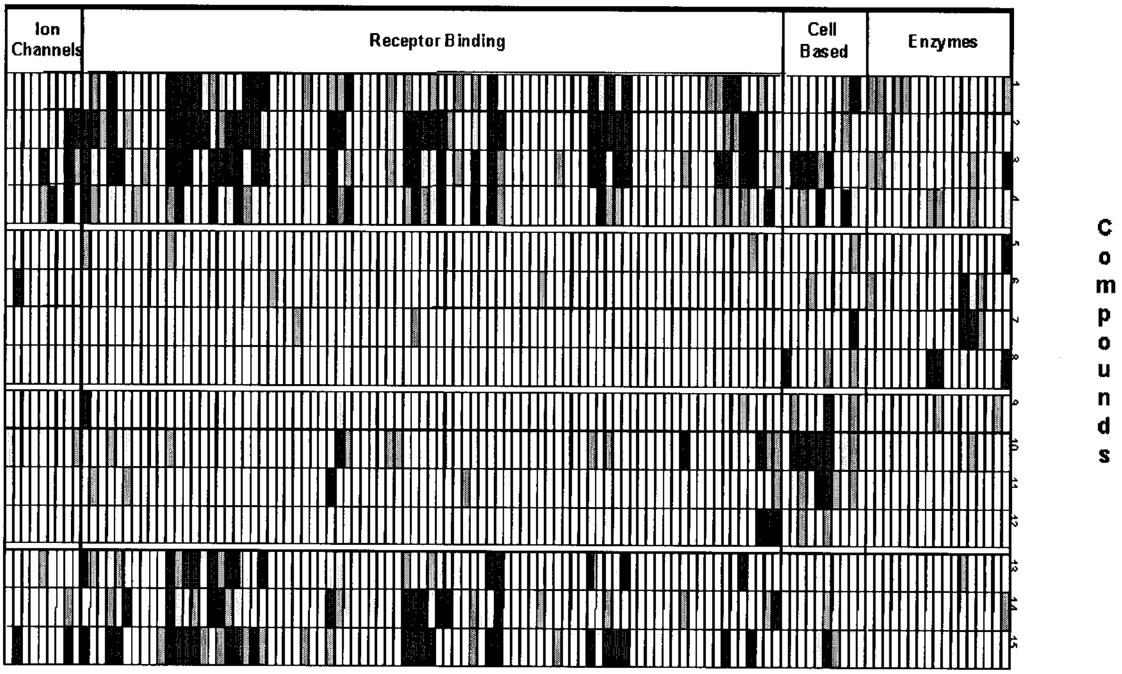

*Figure 2. Activity profiles of compounds against more than 100 di erent targets. The gray scale coding represents the activity obtained in each test, typically expressed as per cent activation or inhibition depending on the target.*

entities that show activity to the respective target group, e.g. G protein-coupled receptors, kinases etc., or on lead explosion by the selection of drugs with a comparable profile obtained in profiling. The feedback of the results to molecular modelling enables the scientist to follow a more rational way of drug design that will help to reduce the effort and time normally spent.

### Conclusion

During the past decade high throughput screening and ultra high throughput screening have significantly contributed to the advances in drug discovery. The increase in associated research costs and the bottlenecks further down the development have increased the demand for alternative approaches. Among these approaches, high throughput profiling combines both the conventional screening strategy combined with data mining technologies that will foster drug discovery in the new millennium.

### Acknowledgements

The outstanding scientific work of Dragos Horvath, John Cargil and Xianqun Wang in designing the database environment and modelling algorithms is greatly acknowledged.

### Reference

1. Prentis, R. A. and Walker, S. R., 1986, Trends in the development of new cardiac medicines by UK-owned pharmaceutical companies (1964–1980). *Br. J. Clin. Pharmacol.*, **21**, 437–443.

